# Supporting a lifetime of fitness for the military veteran athlete: a narrative review

**DOI:** 10.3389/fspor.2024.1510422

**Published:** 2024-12-12

**Authors:** Melissa J. Tinney, Chantal Nguyen

**Affiliations:** ^1^ Department of Physical Medicine & Rehabilitation, University of Michigan Medical School, Ann Arbor, MI, United States; ^2^ Department of Physical Medicine & Rehabilitation, Lieutenant Colonel Charles S. Kettles VA Medical Center, Ann Arbor, MI, United States; ^3^ Department of Orthopaedic Surgery—Division of Physical Medicine & Rehabilitation, Stanford University, Redwood City, CA, United States

**Keywords:** military veteran, physical activity, fitness, adaptive sports, inclusive sports

## Abstract

The military veteran starts their career at peak physical fitness. Once injured or retired, physical activity for the veteran is integral to rehabilitation, recovery, and ongoing wellness. This may require adaptation for continued participation in physical activity. The military veteran, in the United States, has access to resources which can facilitate ongoing physical activity, engagement in competitive and recreational sports, no matter what age or ability. Reviewing the current literature will help understand the scope of programs available, their outcomes, and strategies employed to support a lifetime of fitness that may be applied to other populations and health care systems.

## 1 Introduction

Optimal physical fitness is inherent to the job in the military. Once injured or retired, physical activity can decline for the military veteran. The need to stay active is important, given the multiple comorbidities in this population. Veteran populations have shown to be less healthy than non-veteran populations, self-reporting higher incidence of multiple diseases (1). Veterans in 2019 were estimated to have higher rates of morbidity for obesity/overweight status, heart disease, stroke, cancer, lung disease, arthritis, and diabetes. This has been attributed to stressors of multiple deployments in recent decades (1). According to the National Center for Post-Traumatic Stress Disorder (PTSD), veterans are more likely to have PTSD during their lifetime at a rate of 7%, compared to civilians and veterans receiving care within the United States (2).

A multitude of VA programs for physical activity have been developed. Because of the inclusivity and national reach of these programs, outcomes are studied at a large scale over a diverse population. This literature review aims to identify components of these programs and strategies that are implemented and what areas of health outcomes they impact, which is applicable to the general, non-veteran population interested in maintaining a lifetime of fitness.

## 2 Materials and methods

A literature search was conducted in 3 databases (PubMed.gov, CINAHL via EbscoHost, and Scopus.com) to identify studies regarding physical activity and the military veteran. The search strategies utilized controlled vocabulary and keywords for veterans; keywords for specific programs including Gerofit, Whole Health, VA MOVE!, Golden Age Games, and Wheelchair Games; adjacency searching for exercise across the lifespan, lifetime fitness adaptive physical activity, continuum of fitness, geriatric physical activity, and integrated exercises; and keywords for adaptive sport, inclusive sport, adaptive athletes, winter sports clinic, and summer sports clinic. The searches were not restricted by publication year or language, but were limited to articles published in the United States.

## 3 Results

Searching 3 bibliographic databases and utilizing Covidence software, 316 references were imported and 140 duplicates were removed. Of the 176 studies for title and abstract screening, 126 studies were excluded. 50 studies were assessed for full-text eligibility and 40 articles were included.

The majority of studies ranged from publication years of 2007–2024. Of the 40 included articles, there were 19 on adaptive athletes, 9 on older athletes, 3 on mental health in athletes, 2 on novice athletes, and 7 pilot studies. The majority of studies were either cross-sectional (14/40), qualitative analyses (4/40), retrospective analyses (3/40), or cohort analyses (3/40). Funding was primarily from VA offices and programs, with 25/40 government-funded studies included.

## 4 Programs

As part of the VA's approach to care that supports a veteran's health and well-being, there are a variety of cited VA programs in the literature that facilitate participation in physical activity inclusive of the new, older and/or disabled athlete. Programs include the National Veterans Wheelchair Games (NVWG) (3), National Veterans Golden Age Games (NVGAG) (4), Gerofit (5), National Disabled Veterans Winter Sports Clinic (NDVWSC) (6), National Disabled Veterans Golf Clinic (NDVGC) (7), VA MOVE! program (8), National Veterans Summer Sports Clinic (9) (NVSSC) (Table 1).

**Table 1:** National VA programs facilitating organized participation in physical activity for all veteran athletes, organized by year of inception.

Event	Inception	Location	Qualifying diagnoses	Number of participants (average)	Sports/activities
National Veterans Wheelchair Games (NVWG) (3)	1981	United States (different location each year)	SCI, TBI, amputation, central nervous system (CNS) pathology necessitating use of wheelchair (i.e., multiple sclerosis, cerebral palsy)	550	Wheelchair basketball, quad rugby, power soccer, throwing events, cornhole, disc golf, handcycling, swimming, adaptive fitness, 9-ball, softball, table tennis, wheelchair slalom, motor rally, boccia, pickleball, bowling, air pistol, air rifle, bass fishing
National Veterans Golden Age Games (NVGAG) (4)	1985	United States (different location each year)	Age ≥50 years old	>900	Basketball, cycling, pickleball, power walking, swimming, track & field, badminton, bowling, disc golf, table tennis, pistol, air rifle, basketball free-throw, boccia, cornhole, nine ball, shuffleboard
Gerofit (5)	1986	Founded in Durham, North Carolina, but now in 33 VA systems	Age >65 years old, stable medical condition and independent with ADLs	Thousands	Individual and group supervised exercise sessions, including core strengthening, balance, tai chi, dance, treadmills, ellipticals, stair climbers, cycling
National Disabled Veterans Winter Sports Clinic (NDVWSC) (6)	1987	Snowmass, Colorado	SCI, TBI, amputation, visual impairment	400	Adaptive sled hockey, adaptive Alpine/Nordic skiing, snowmobiling, fly fishing, curling, scuba diving, rock climbing
National Disabled Veterans Golf Clinic (NDVGC) (7)	1994	Riverside, Iowa	SCI, TBI, amputation, CNS pathology, visual impairment	200	Adaptive golf, air rifle, bicycling, bowling, and kayaking
VA MOVE! Program (8)	2008	Nationwide	BMI of 30 kg/m^2^ or BMI 25–30 kg/m^2^ with metabolic syndrome	>35,000	150–300 min of physical activity per week
National Veterans Summer Sports Clinic (NVSSC) (9)	2008	San Diego, California	SCI, TBI, amputation, visual impairment, burns, MS, stroke, depression, PTSD	150	Cycling, surfing, sailing, adaptive fitness, yoga, kayaking, archery, and pickleball

For the individual veteran, the VA covers prescription of different types of adaptive sports equipment with a qualifying impairment and appropriate medical justification. Other VA grants provide a stipend for those training with a Paralympic or Olympic team to facilitate participation at the national/international level (10). There is also a VA Adaptive Sports Grant Program that provides funds for qualifying organizations to organize and sustain longitudinal adaptive sports opportunities for veterans with disabilities (11). One study looking specifically at the characteristics of community programs showed 76% of programs reviewed have VA medical center affiliation (12).

In addition to these large, national organized events, the VA has offered smaller local adaptive sports events. One example in the literature is, Heroes on the Hudson, an annual one-day adaptive kayaking and sailing event for those with psychological (PTSD, depression), physical (such as amputees, spinal cord injury, traumatic brain injury), and visual impairments. One pilot program was based on the framework of Gerofit, to prepare frail older veterans for surgery, as part of a prehabilitation program (13). One pilot program was based on the framework of Gerofit, to prepare frail older veterans for surgery, as part of a prehabilitation program (14). Another pilot program was created at the War-Related Illness and Injury Study Center at the VA New Jersey Health Care System to address Gulf War Illness. The study's authors note “this is the first clinical program that has piloted an intensive interdisciplinary and integrative functional medicine-based virtually delivered Whole Health coaching program in Veterans with complex post-deployment chronic multi-symptom illness from deployment- related exposures”. It was described as a 6-month video-to-home telehealth program including: functional medicine assessments, individual and group nutritional and adaptive exercise coaching with portable exercise equipment, group mindfulness meditation and yoga, guest health lectures, character strength evaluation and coaching, and targeted nutritional supplementation that were tailored to each Veteran (15).

Because of the known comorbidity of PTSD, other pilot studies focused on providing exercise and physical activity to improve mental health. One pilot program studied the use of group integrative exercise to reduce symptoms of PTSD. Weekly 1-h exercise sessions included aerobic exercise, strength training with weights and resistance bands, and yoga movements and poses presented within a framework of mindfulness principles, with one principle presented in each session as the focus of the week (16). Another pilot study looked at ways to improve health and wellness for veterans in the Mental Health Intensive Case Management Program, a community-based intensive program for veterans (involving walking intervention as modified from the MOVE! program, weekly in-person sessions with trained mental health providers) with severe mental illness (SMI) who are at risk for decompensation and frequent hospitalizations (17).

There are many non-VA programs that promote physical activity for veterans (11), but few were found in this literature review. The U.S. Paralympic Military Sport Camp (USPMSC) and Higher Ground, an adaptive outdoor recreation program are noted (18, 19). At the international level, the annual Invictus Games involves disabled veterans from around the world, including the United States, to compete in various sports over the course of one week (20).

## 5 Outcomes

For the veteran athlete, participation in organized programs for physical activity and adaptive sports has led to comprehensive improvements in physical health, psychological health, and overall quality of life (QOL)/well-being.

### 5.1 Health parameters outcomes

Veteran participation in the VA MOVE! program has led to increased overall weight loss, presumably with improvements in both diet and physical activity parameters (21). Another study looked at medication prescriptions. After 1-year of participation in Gerofit, participants showed when comparing prescriptions filled in the pre-Gerofit baseline to the post-12-month period, 55% of patients had a decrease in their overall number of fills of medications for multiple comorbidities, which included opiates, cardiac, mental health, diabetes, and lipid lowering medications (22). Blood pressure (BP) and weight were also studied in community-based programs and within 2 months of participation BP and average weight decreased in one study. The data reported in that study showed the mean weight of participants decreased by 9 lbs, percent of controlled BP increased by 24, and percent of uncontrolled BP decreased from 40% to 16% of participants (21). BP and weight improvements were also seen in community-based programs and within 2 months of participation blood pressure and average weight decreased. The mean weight of participants decreased by 9 lbs, percent of controlled blood pressures increased by 24, and percent of uncontrolled blood pressures decreased from 40% to 16% of participants (23).

### 5.2 Fitness and mobility outcomes

One study of obese individuals demonstrated they make improvements and show clinically significant changes in performance measures of mobility compared with overweight and normal-weight individuals (24). Similarly, veterans with SMI, participating in Gerofit, also make improvements in mobility, as well as strength and endurance, similar to veterans without SMI, showing the capacity for improvement with complex comorbidities (25). In frail elderly veterans, a pre-surgical exercise program demonstrated improvement in fitness outcomes: gait velocity, chair stands, 6-minute walk, 8 foot up and go, and arm curls (14).

Partaking in the NVWG significantly increased veterans' wheelchair mobility when compared to mobility at home or in their local communities, with increased distance traveled (4,466.2 vs. 1,367.4 m), wheelchair propulsion velocity (0.76 vs. 0.64 m per second), and continuous drive time (5.2 vs. 2.5 min). Furthermore, wheelchair users at the NVWG had fewer stops every 500 m while at the NVWG when compared to being at home. This included improvements in the same movement parameters for power wheelchair users during the NVWG (26).

### 5.3 Psychological health outcomes

Physical activity has been incorporated into treatment of PTSD, as shown in a VA pilot study. In one particular study, the integrated exercise group demonstrated greater improvement in PTSD symptom severity and psychological QOL compared with participants randomized to the wait list control group (16). One group assessed Gerofit data and noted significant improvement in their overall PTSD symptoms as well as each of the four PTSD symptom clusters (intrusion, avoidance, negative cognitions/mood, hyperarousal) after three months (27). A randomized control trial of a 12-week integrative exercise program for war veterans with PTSD saw significant improvements in mindfulness, interoceptive bodily awareness, and positive states of mind compared to a wait list control. These changes in secondary outcomes may be partial mechanisms of action for how integrative exercise creates the observed improvements in PTSD symptoms and QoL (28). In a specific non-VA program, Higher Ground, they performed a non-randomized experimental trial to study the impact of a sports and recreation program on veterans with PTSD symptoms. Participants showed reduction in PTSD symptoms after participants completed the program (29). After participation in Higher Ground events, veterans noted decreased rates of anger and depression, instead highlighting a newfound sense of energy and motivation to participate in the sport and within the community (19). These same improvements are seen on the local and international level with the Invictus Games (20). Even with a 1-week event, the NVSSC, one study showed depression, anxiety, social functioning, and positive and negative affect significantly improved from pre-to post program, but returned to baseline levels at 3-month follow-up (30). In a secondary analysis of the same group, there were significant improvements in depression, generalized anxiety, insomnia, positive affect, and negative affect immediately following the NVSSC, as well as improvements in depression anxiety and positive affect immediately following a singular session activity. Veterans with probable PTSD also reported significant reductions in PTSD symptom severity over the course of the program, which were reliable and statistically significant (30).

### 5.4 Quality of life and social outcomes

From a QOL perspective, veterans participating in wheelchair sports report increased overall life satisfaction with a predominant increase in social network or number of friends, which was highlighted by 98% of 132 participants at the 26th NVWG and 20th NDVWSC (31). Longer duration of participation in physical activity (at least ten years) led to overall higher self-esteem scores when compared to more novice veterans participating in physical activity for less than 5 years, in addition to improvements in self-efficacy/ability to independently perform activities of daily living (32). In general, veterans participating in the NVWG report a higher quality of life and improvements in relationships with the community, as formally noted with documented higher Sports Participation Outcome Research Tool and Comprehensive Uniform Surve (SPORTACUS) and Functional Mobility Assessment (FMA) scores (33). These psychological benefits and improved social support have yielded further communal benefits, with increases in employment rates, after participation in the NVWG. Of note, there is a correlation of increased employment rates with additional years of adaptive sports participation; veterans attending at least 3–4 NVWG's noted positive improvement in ability to obtain employment (34). In a similar light, participation in the USPMC led to improvements in perception of a veteran's disability, thus helping him/her focus on optimism, autonomy, inspiration to pursue other organized recreational adaptive sports, and desire to engage in pre-injury interests (18).

Veterans' perspectives on participation in Gerofit identified factors that could be viewed as facilitators. Amongst participants (less than 3 months gap in exercise) and non-participants (more than 3 months gap in exercise), camaraderie was uniformly noted as a valuable part of the Gerofit program (35). Social connectedness was rated as high by all Gerofit participants, with the majority endorsing positive social support and relatedness with their exercise companions. In those veterans with PTSD that reported symptom improvement, positive feelings of social connection were significantly related, accounting for approximately 20% of the gain (27). Morey and colleagues did a 5-year retrospective analysis of Gerofit and noted it has several components that particularly enhance the psychosocial aspects of the program, particularly the program is for veterans who share a strong common bond (36). In a study looking at older veterans with SMI, participating in Gerofit, those who were retained at 6 months had better health-related QOL (25).

## 6 Discussion

**Figure 1:**
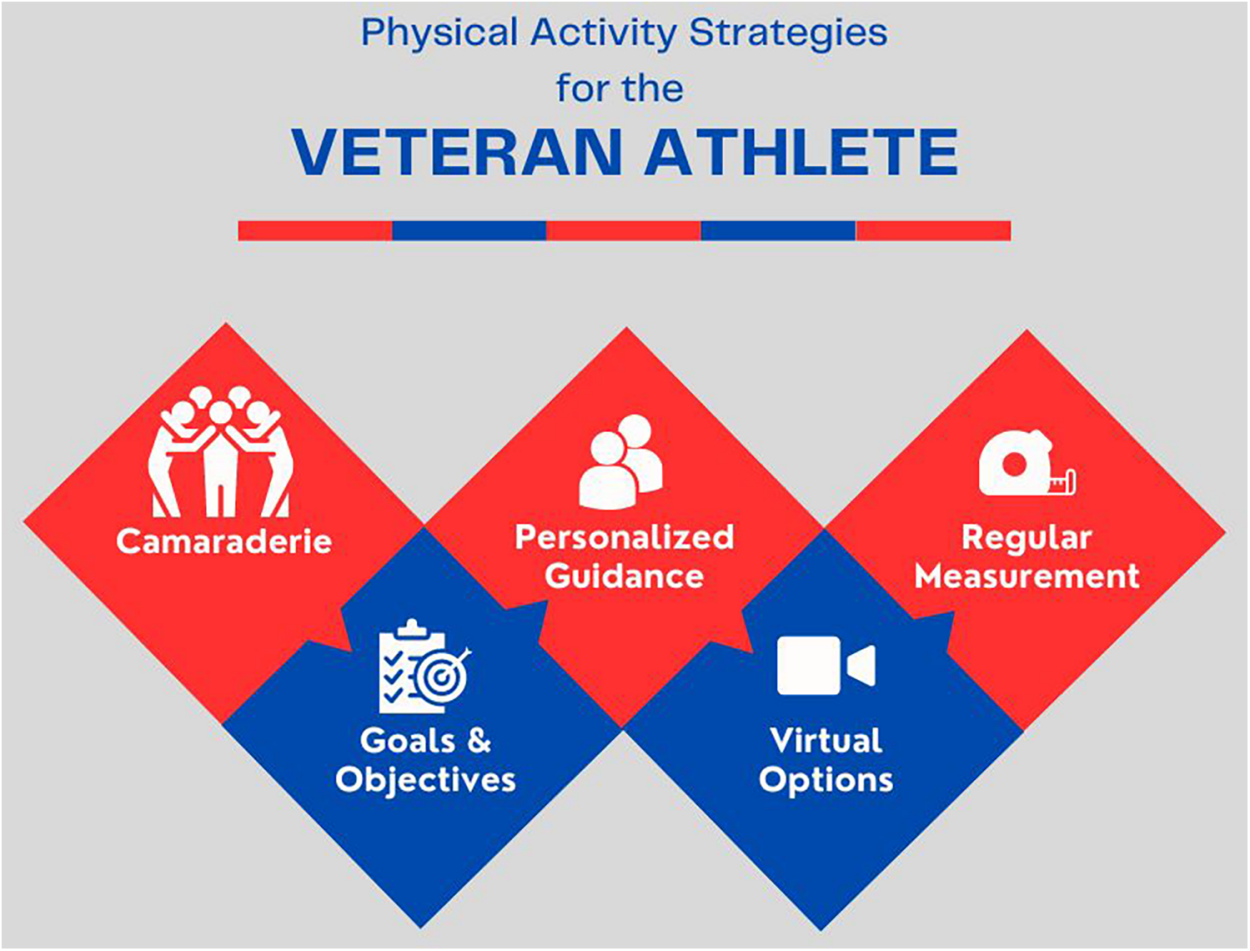
Physical activity strategies for the veteran athlete..

The VA has built a uniquely robust and inclusive infrastructure to support physical activity for all ages and abilities and has established a framework to analyze the efficacy of such wide-reaching programs. The individualized, whole health approach has demonstrated positive outcomes, with proven success utilizing key strategies across these programs. These key strategies include camaraderie, defined goals and objectives, personalized guidance, virtual platform options, and measurable outcomes (Figure 1).

Despite positive outcomes from these key strategies, veteran athletes still face known barriers to participation in exercise and sports, including transportation and travel distance (37). To address this barrier, the VA has created pilot programs to explore increased telehealth services or implementation of modified virtual programs, such as the Gerofit program. Changes in the program, including modifying performance measures adjusted to a smaller space and using only mobile equipment and plyometric (weight-bearing) exercises made programs like Gerofit accessible to rural populations (37). A systematic review was also conducted specifically looking at telehealth or virtual delivery of the Gerofit program and found several studies showing similar gains in physical health outcomes from virtual vs. in-person exercise (38).

VA programs emphasize an individualized approach, from the novice to elite athletes and provide outcomes in populations that would normally be excluded from exercise intervention trials (24). As referenced from Browne and colleagues, goal setting and motivational strategies, with examination of mechanisms of change within multicompartmental exercise programs like Gerofit, can specifically yield beneficial physical activity outcomes (39). This demonstrates that veterans, even with complex conditions, can benefit from supported and strategic ongoing physical activity. Furthermore, veterans who find difficulty integrating into their communities or sustaining a job due to their medical history or impairments have found improvement in social connection with increased participation in physical activity, which extends to improvements in self-efficacy and higher rates of sustaining employment (34).

This review demonstrates a limited amount of published information across all outcomes for both VA and non-VA programs. Research on these programs are mostly pilot studies or singular year outcome analyses, which limits generalizability of results and determination of long-term effectiveness of each program. Future research may examine longitudinal health outcomes given the longevity of these programs over all military service eras. There is an opportunity to cumulatively study at least tens of thousands of veterans participating in these programs in a strategic, systematic way via fellowships, quality improvement programs, and/or research centers.
